# Evaluating hazard, vulnerability, and capacity through local knowledge for volcano risk reduction

**DOI:** 10.4102/jamba.v17i1.1876

**Published:** 2025-09-30

**Authors:** Pipit Wijayanti, Rita Noviani, Sorja Koesuma, Yunus A. Wibowo, Anang W. Nirwansyah, Puspita I. Wardhani, Siti H. Hafida, Sriyanto Sriyanto, Ana Andriani, Fathi Muzaqi

**Affiliations:** 1 Department of Geography Education, Faculty of Teacher Training and Education, Sebelas Maret University, Surakarta, Indonesia; 2 Disaster Research Centre, Sebelas Maret University, Surakarta, Indonesia; 3 Department of Physics, Faculty of Mathematics and Natural Science, Sebelas Maret University, Surakarta, Indonesia; 4 Department of Geography Education, Faculty of Teacher Training and Education, University of Muhammadiyah Surakarta, Surakarta, Indonesia; 5 Centre for Disaster Mitigation Study, University of Muhammadiyah Surakarta, Surakarta, Indonesia; 6 Department of Geography Education, Faculty of Teacher Training and Education, University of Muhammadiyah Purwokerto, Banyumas, Indonesia

**Keywords:** local spatial knowledge, Merapi, participatory geographic information system, disaster risk reduction, hazard, vulnerability, capacity

## Abstract

Indonesia has a high risk of geological disasters because its location is on the arc of plate movement. One of the main threats comes from Mount Merapi, an active volcano that often erupts, significantly impacting the surrounding community. This study uses the Participatory Geographic Information System (PGIS) approach to assess the community’s hazard, vulnerability and capacity on the slopes of Mount Merapi, Magelang. This locale-based mapping integrates three main variables to provide a more comprehensive risk picture. The results show that 80% of households are in the high to very high hazard category, with the main threat types being volcanic ash and hot clouds. Household vulnerability varied, with 44.4% in the low category and 16% in the high category, indicating the presence of a significant population that was particularly vulnerable. On the other hand, the capacity of the community is still unevenly distributed, with almost half of the population having a very low capacity. Local Spatial Knowledge (LSK) is essential in reducing disaster risk, including public understanding of natural signs, safe evacuation routes and tradition-based mitigation strategies.

**Contribution:**

The integration of LSK with Geographic Information System (GIS) technology strengthens the effectiveness of risk assessment, allowing for more accurate mapping and targeted intervention strategies. Public awareness of risks has grown significantly because of greater access to information enabled by digital technology, although local values still need to be preserved.

## Introduction

Indonesia is a country that has a high risk of geological disasters because its location is in the arc of plate movement (Bemmelen & Van Reinout 1970). Indonesia has more than 127 active volcanoes, one of which is Mount Merapi. Mount Merapi, one of the most active volcanoes in the world, poses a significant danger to the surrounding communities, especially in Central Java (Chan, Konstantinou & Blackett 2021; Wigati et al. 2023). Known for its frequent eruptions, Mount Merapi’s activity has increased in recent decades, culminating in several significant eruptions. The eruption in 2010 was one of the most devastating in Indonesia’s recent history (Chasanah & Sakakibara 2022), affecting areas such as Magelang, Boyolali, Klaten and Sleman Regencies, and causing more than 300 deaths and displacing hundreds of thousands of people (Pallister et al. 2013; Surono et al. 2012). These eruptions highlight the destructive nature of volcanic hazards, which could lead to immediate and long-term impacts on physical infrastructure, social stability and economic well-being. Direct effects include loss of life and extensive property damage, while indirect impacts are seen on disrupted livelihoods, reduced economic activity and long-term ecological damage. Given these risks, disaster preparedness is essential, especially in areas with high exposure to natural hazards (Meilani & Hardjosoekarto 2020). The need for proactive risk management becomes more apparent as communities seek to minimise the impact of future eruptions.

The community has lived around Mount Merapi for a long time and understands the conditions and characteristics of its environment (Voight et al. 2000). Therefore, further studies should emphasise socio-cultural approaches that highlight the role of community-based knowledge and behaviour (Donovan Suryanto & Utami 2012). Until now, most studies on Mount Merapi have focused on physical hazard characteristics such as lava flow modelling and pyroclastic flow behaviour (Charbonnier & Gertisser 2012; Damby et al. 2013; Thouret et al. 2000). Meanwhile, social studies have examined community vulnerability using tools such as the Social Vulnerability Index (Siagian et al. 2014), or explored evacuation effectiveness (Jumadi et al. 2018; Kurniawan et al. 2021; Rindrasih 2018). Understanding this local space is key to reducing disaster risk, including through local practices. One of the essential components in risk management is Local Spatial Knowledge (LSK), which includes people’s understanding of the environment and the specific hazards that exist (Cadag & Gaillard 2012; Mercer et al. 2012; Šakić Trogrlić et al. 2019). In addition, incorporating LSK into disaster management efforts fosters trust and cooperation between the community and external stakeholders. However, very few studies have explicitly integrated local knowledge as a core analytical framework for assessing risk and developing mitigation strategies.

This article aims to explore the approach of LSK to assess hazards, vulnerabilities and capacities and integrate them into geographical information systems (GISs) to visualise and understand the spatial understanding of communities in disaster-prone areas of Merapi. When communities see their knowledge valued and utilised in formal planning and mitigation strategies, they are more likely to participate actively in preparedness activities. This collaborative approach empowers communities and increases the effectiveness of risk-reduction initiatives. For example, citizens may be more willing to comply with evacuation orders or participate in disaster drills if these actions are consistent with their knowledge and experience. In addition, this collaboration can be a model for other regions facing similar challenges, demonstrating the importance of cultural understanding in managing natural hazards.

## Literature review

### Local spatial knowledge

Local Spatial Knowledge refers to collective wisdom and experience passed down from generation to generation, often transmitted through oral traditions, folklore, legends and cultural practices (Kheladze, Mccall & Van Westen 2011). In the context of Merapi, this knowledge includes the public’s understanding of warning signs from nature, safe and hereditary evacuation routes and high-risk areas based on historical eruptions. Local experience is invaluable, as it allows communities to navigate and respond to disaster threats with familiarity and insight that may not be captured in conventional scientific assessments. Kuhn & Duerden (2021) describe LSK as a ‘home and space of action’, which shows an inherent and ongoing awareness of the soil and its dangers. By recognising urgent issues and encoding this information in terms that are understandable to the community, LSK provides a foundation for disaster risk management that is culturally and practically relevant.

In the context of disasters, LSK is vital in assessing hazards, vulnerabilities and capacities more accurately through community understanding (Giordano, Preziosi & Romano 2013; Kienberger 2012; Peters-Guarin, McCall & Van Westen 2012). Local spatial knowledge encourages individuals and groups to identify high-risk areas, recognise safe zones and implement timely evacuation strategies (Paudel et al. 2024; Vasileiou, Barnett & Fraser 2022). In addition, this knowledge fosters resilience by equipping communities with the tools to anticipate danger and adjust their behaviour. Unlike purely mathematical or scientific models that primarily emphasise physical risk indicators, LSK offers a more holistic view by incorporating social and cultural dimensions (Briones et al. 2023, 2024; Gislason et al. 2011; Iervolino et al. 2024; Manta et al. 2021; Nakamura et al. 2008; Scaini et al. 2014; Taher, Pandey & Kumar 2025), which may focus heavily on physical risk factors, LSK provides a holistic view of regional risk in terms of social and cultural dimensions. Through LSK, it not only raises community situational awareness but also assists external stakeholders, such as government agencies and disaster management organisations, in designing response strategies that align with local values and behaviours (Barclay et al. 2022; Burningham, Fielding & Thrush 2008; Kjellgren 2013; Macnight Ngwese et al. 2018; Nirwansyah et al. 2024). This knowledge can be crucial for local leaders and policymakers when making quick and informed decisions in the face of imminent volcanic activity, as it complements scientific data with first-hand empirical insights.

The integration of LSK into GIS strengthens its usefulness, providing a visual representation of hazard, vulnerability and capacity data that combines human, economic and environmental elements (Zandlová, Skokanová & Trnka 2023). When combined with LSK, GIS mapping allows for a more comprehensive risk assessment by layering scientific data with the spatial knowledge of the community. This integrated approach helps identify areas that need urgent attention, improves the accuracy of hazard maps and supports efficient resource allocation for disaster preparedness and response. According to Van Westen (2013), incorporating LSK and GIS technologies strengthens local disaster governance and enables relevant authorities to respond more effectively. Previous research on LSK includes the use of Participatory Geographic Information System (PGIS) for participatory spatial planning and good governance (McCall & Dunn 2012), assessment of agricultural services in rural areas (Debolini et al. 2013), integration of community vulnerability mapping for disaster risk management in the Caribbean (Canevari-Luzardo et al. 2017), ethnography and LSK to describe the Duero River border landscape (Hearn 2021).

### Hazard

A hazard is a natural event or human activity that has the potential to cause loss or disturbance to life and the environment (Hait & Sahu 2024). In the context of natural hazards, hazards include events such as earthquakes, volcanic eruptions, floods and tsunamis. Hazard measurement is crucial because it is the main component that determines the level of risk (Kurniawati et al. 2023). Important characteristics of hazards include the type, time of occurrence, depth and duration, frequency, triggering factors and impacts (Taştan & Aydinoğlu 2022).

In LSK’s study of volcanic disasters, hazards are divided into primary and secondary hazards. Primary hazards include lava melt, pyroclastic flows and toxic gases that appear immediately during an eruption (Farquharson & Amelung 2022). Meanwhile, secondary hazards include rain-fed lava, flash floods and landslides that occur because of the continuation of volcanic activity (Italiano 2023). Local knowledge plays an important role in recognising the timing and frequency of hazard events, usually passed down from generation to generation and used in traditional early warning systems.

Hazard triggers are differentiated into natural ones, such as magma activity, tectonism and high rainfall, as well as anthropogenic ones, such as mining and settlements in vulnerable zones, which can accelerate or exacerbate volcanic activity. Hazard impacts are divided into primary impacts (physical damage such as destruction of buildings and agricultural land) and secondary impacts (health crises, socio-economic disturbances and environmental damage). Public understanding of various aspects of hazard is an important part of LSK and plays a key role in community-based disaster risk reduction strategies.

### Vulnerability

Vulnerability is an important component in disaster risk measurement that reflects the extent to which a system, community or individual is exposed and unable to cope with the impact of a hazard (Birkmann & McMillan 2020). Vulnerabilities arise from factors such as poverty, low access to information, socio-political inequality and a lack of infrastructure, which are exacerbated by dynamic pressures and insecure conditions (Zarowsky, Haddad & Nguyen 2013).

In the context of volcanic disasters and the LSK approach, vulnerability encompasses three main aspects: physical, social and economic. Physical vulnerability reflects residential conditions and people’s ability to adapt to disaster-prone environments. Social vulnerability is related to demographic and health structures, where vulnerable age groups and people with certain diseases are more at risk of being affected by volcanic disasters. Meanwhile, economic vulnerability is measured through income stability and people’s types of livelihoods, which determine how much of an economic impact is borne in the event of a disaster.

### Capacity

Capacity refers to the ability of a society or system to anticipate, respond and recover from the impact caused by hazards (Gaillard, Cadag & Rampengan 2019). This capacity can include various aspects of resources, including physical, social and economic resources (Dharmadasa et al. 2023). Physical resources include the condition and characteristics of residential buildings and their maintenance processes in the face of potential disasters. Social resources relate to the ability of communities to build social protection systems, including access to health insurance and community support. Meanwhile, economic capacity is reflected in the ability of people to have savings or reserves of financial resources as a form of anticipation of the impact of disasters.

In the context of LSK, community capacity is measured based on the level of experience and local understanding in preparing for disaster hazards. Local knowledge that is passed down from generation to generation, as well as experience in dealing with previous disasters, is an important part of this capacity. The higher the level of capacity that the community has, especially in terms of preparedness and adaptation to risks, the lower the potential risks and impacts that can be caused by disasters.

## Research methods and design

### Overview study area

Mount Merapi is located in the Special Region of Yogyakarta (Sleman Regency) and the Province of Central Java (Klaten, Magelang and Boyolali Regencies) as shown in Figure 6. The mountain is 2930 m above sea level and was formed about 400 000 years ago during the Quaternary period, part of the Cenozoic era, the Middle Pleistocene period, because of the interaction of the Indo-Australian plates.

Mount Merapi has explosive and non-explosive eruption types, depending on the viscosity of magma and the pressure of volcanic gases (Andreastuti, Alloway & Smith 2000; Camus et al. 2000; Newhall et al. 2000). With an andesite-dacitic type of magma, the mountain often forms lava domes at risk of collapsing, producing hot clouds or locally known as ‘*wedhus gembel*’. As the most active volcano in Indonesia, its eruption is characterised by pyroclastic flows and destructive lava.

Although dangerous, the fertile land around Mount Merapi is an attraction for residents who depend on its natural resources for their livelihoods. This makes Mount Merapi one of the most densely populated volcanoes in the world. As a result, this area’s vulnerability level is very high, so adequate community preparedness is needed. This research was conducted on the slopes of Mount Merapi, Magelang Regency, Central Java Province, Indonesia.

### Method: Local spatial knowledge and hazard, vulnerability, capacity

Hazard, vulnerability and capacity mapping were used in the PGIS method. This method maps communities, households, individual priorities and public perceptions. Participatory Geographic Information System encourages community engagement and enables local knowledge-based assessment of phenomena that outsiders can access (Brown & Fagerholm 2015; Dunn 2007; Jankowski 2009). The mapping results using PGIS through a data plotting system are integrated with an Environmental Systems Research Institute GIS with ArcGIS Pro software.

Hazard, vulnerability and capacity assessments are carried out by collecting information based on local community understanding (Table 1). The sample in this study was selected using the stratified sampling method, in which the population was divided into three strata based on disaster-prone zones: levels 1, 2 and 3. From each stratum, respondents were proportionally selected according to the number of households in each zone. A total of 90 household respondents were selected 30 from Zone 1 (low risk), 30 from Zone 2 (moderate risk) and 30 from Zone 3 (high-risk). The criteria for respondents included being permanent residents who have lived in the area for an extended period and being at least 40 years of age. This approach ensured a balanced and contextually relevant representation based on local experience in facing disaster risks. This study uses Disaster Prone Areas (KRB) as the unit of analysis, based on the disaster-prone zoning classification issued by the Volcanological Survey of Indonesia. Disaster-prone areas are areas that are identified as having high disaster potential. According to Regulation No. 11 of 2016, the Merapi KRB is divided into three zones: KRB I (low risk), KRB II (moderate risk) and KRB III (high-risk).

**TABLE 1:** Definition variable of hazard, vulnerability and capacity.

Variable	Type
Hazard	Type hazard
	When disaster occurs
	Depth of disaster
	Frequency of disasters
	Driving factors
	Impact
Vulnerability	Economic vulnerability
	Social vulnerability
	Structural vulnerability
Capacity	Economic capacity
	Social capacity
	Structural capacity

*Source:* Adjusted from Kheladze, N., Mccall, M. K. & Van Westen, C. J., 2011. ‘Assessing the feasibility of using local spatial knoweldge in disaster risk management in georgia’, Thesis, International Institute for Geo-Information Science and Earth observation (ITC) Enschede, The Netherland

The identification of local spatial understanding of the Merapi volcanic disaster was carried out by an interview process with 90 households, including local geographic knowledge, experience and observation, local practices and tradition, social connectivity, environmental adaptation and integration of modern technology. The data were tabulated and analysed using quantitative graphs of interview data.

In the Merapi area, the goal is for the selected respondents to understand the surrounding conditions. Hazard, vulnerability and capacity assessments are conducted based on the following variables:

The assessment was carried out with a grouping approach that integrated three leading indicators – hazard, vulnerability and capacity – to provide a comprehensive picture of the level of disaster risk in the analysed area. Each variable on these three indicators is classified into four main categories: very low, low, high and very high. This grouping aims to identify areas with diverse risk characteristics so that mitigation measures can be adjusted to local conditions.

Each variable is normalised using a maximum value of 4, representing the highest level of danger, vulnerability or capacity. This normalisation provides consistency in assessment, facilitates comparisons between regions and improves accuracy in prioritising interventions. The assessment process is carried out by calculating each variable using a formula designed to ensure the accuracy and consistency of results. This formula takes into account the weight of each indicator so that the total value can reflect the level of risk proportionally to the formula as follows (Equation 1):


$$Zi=\frac{xi-min(x)}{max(x)-min(x)}$$ [Eqn 1]

where *Zi* is a variable score for each household, *min(x)* indicates the minimum variable score of all households, and *max(x)* indicates the maximum variable score of all households. The variable values are grouped into four categories: 1 (very low), 2 (low), 3 (high) and 4 (very high). This classification represents hazard levels, vulnerabilities and capacities with quantitative symbols on hazard, vulnerability and capacity maps.

### Ethical considerations

Chairperson of the Research Ethics Commission, after a series of discussions and assessments, hereby grants permission to Dr. Pipit Wijayanti, S.Si., M.Sc., a lecturer in Geography Education at the Faculty of Teacher Training and Education, Universitas Sebelas Maret, to conduct research and has passed the Research Ethics Commission with number 10/UN27.02.11/PP/EC/2024, dated 26 September 2024.

## Results

### Local spatial knowledge

The research revealed a high level of community preparedness in the high-risk area of Magelang near Mount Merapi. A vast majority of respondents (99%) demonstrated local geographic knowledge, with only 1% lacking such understanding.

This local geographical understanding is related to community knowledge of the location of disaster risk areas, evacuation routes, disaster-safe areas and community preparation to face the Merapi eruption. Those familiar with the route demonstrate detailed knowledge, identifying locations such as village halls, schools, village main roads and other strategic points such as gathering places or evacuation points. Most respondents also recognised rivers and slopes as natural and safe paths. This diversity in route selection reflects the adaptation of local knowledge to volcanic risks, demonstrating the readiness of communities beyond route awareness to incorporate adequate geographical understanding to achieve safety.

Experience and observation are essential indicators in assessing the level of local spatial understanding of the community. This aspect reflects the extent to which the community remembers the eruption event, recognises the frequency of eruptions, understands the signs of potential eruptions, and uses this knowledge as a basis for decision-making in the evacuation process. As many as 92% of respondents in the Merapi area indicated that they actively made observations and applied the results of these observations to deal with the threat of eruption. They were able to recall a series of major events such as the eruptions of 1968, 1996, 2004 and 2010. In addition, the community consistently observes natural phenomena that are markers of eruptions, such as an increase in air temperature, rumbling sounds from the top of Merapi, and animal behaviour of descending from the upper slope to lower areas. This suggests that experience and observation are important components of LSK, which is still strongly maintained in the daily lives of the people of Merapi and actually contributes to increasing vigilance and preparedness for disasters.

As many as 63% of respondents no longer apply local cultural practices and traditions. One of the traditions that used to be very well known and often practiced by the people of Merapi is ‘kenduren’ or ‘selametan’, which functions as a plea for salvation and an expression of gratitude. Based on the investigation results, people rarely carry out these traditional practices. On the other hand, the forms of preparedness that are more widely carried out today tend to focus on practical things, such as preparing important documents for evacuation purposes. These practices demonstrate a strong understanding of logistical preparedness for rapid evacuation during emergencies. This local knowledge-based readiness is aligned with a disaster risk mitigation approach that prioritises adaptation to social and geographical conditions. This adaptation provides an overview of how people utilise rapid response strategies to minimise the risk of losing valuable assets during evacuation.

The community has undertaken adaptation to the environment; approximately 64% of respondents reported changes in their daily activities in response to the impacts of the Mount Merapi eruption, particularly after the major eruption in 2010, mainly in agricultural and livestock management. For example, some farmers increase fertilisers to restore soil fertility from volcanic ash, while some use more fermented feed to secure food supplies during emergencies. A portion of the community (28%) reported no significant change in the impact of the Merapi eruption, suggesting varying levels of adaptive capacity among the local population.

Social connectivity is important in maintaining the sustainability and dissemination of LSK. As many as 48% of respondents are actively involved in disaster organisations, strengthening information networks between citizens. In addition, the process of intergenerational knowledge transfer continues informally through daily habits, such as the delivery of evacuation routes and experiences of dealing with eruptions, which strengthen people’s collective memory of disaster risk. In terms of preparedness, access to the fastest information – or rather, say fast, clear and accessible information – is crucial to reducing disaster risk. Around 52% of respondents obtained information from local officials, official institutions such as the Geological Disaster Technology Research and Development Center (BPPTKG), the Regional Disaster Management Agency (BPBD), the Meteorology, Climatology and Geophysics Agency (BMKG), and the mass media. These findings show that the social connectivity of the Merapi community is formed from a combination of community interaction and connectivity with formal institutions, which strengthens the community’s capacity to deal with disasters. A total of 77% of respondents in household communities have integrated modern technology in disseminating information, such as using modern devices, including smartphone applications, WhatsApp groups, social media and radio (HT) to access the latest updates on Mount Merapi’s condition. Meanwhile, 23% still rely on direct information from local authorities or other non-digital sources. Local spatial knowledge reflects the community’s complex understanding of its environmental conditions. This understanding serves as an essential foundation for determining community-based management strategies, particularly in the context of disaster risk reduction.

### Hazard, vulnerability and capacity

**FIGURE 1:**
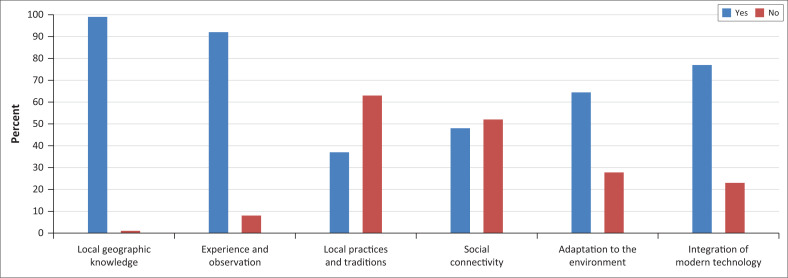
Local spatial knowledge.

The households in the study area were analysed to assess threats, vulnerabilities and capacity to deal with disasters in the Mount Merapi area. The results of this assessment help identify the distribution of risks in various KRB, which are classified into the categories ‘Very Low’, ‘Low’, ‘High’ and ‘Very High’, and presented in Figure 1.

The analysis results are presented in Table 2, which outlines the percentage of households based on the classification of threat value, vulnerability and capacity. In addition, the visualisation map also provides an overview of the spatial distribution for each aspect of the study area.

**TABLE 2:** Value classification of the household.

Category	Hazard	%	Vulnerability	%	Capacity	%
Very low	0	0.0	27	30.0	10	11.1
Low	10	11.1	40	44.4	33	36.7
High	45	50.0	16	17.8	26	28.9
Very high	35	38.9	7	7.8	21	23.3

Table 2 shows the household values based on three primary parameters: hazard, vulnerability and capacity. The hazard distribution is mainly concentrated in the high category, 88.9%. This illustrates the threat most of the population faced on the slopes of Merapi. In contrast, only 11.1% of households were categorised as low hazard, and none were in the very low hazard category. Exposure to disaster threats depends on the type of hazard, time of occurrence, depth, frequency of disasters, driving factors and their impact.

**FIGURE 2:**
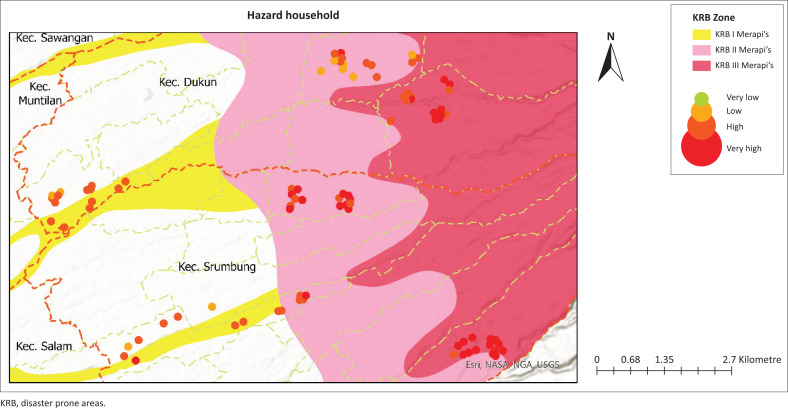
Hazard household mapping.

As shown in Figure 2, the distribution of households shows a tendency to have a perception of a high level of danger, based on various variables that have been measured.

The high hazard level in the Mount Merapi area is influenced by the depth and frequency of disasters, which are strongly inherent in the community’s memory. Almost all respondents stated that all human activities will stop during a disaster. This reflects the depth of the disaster, where the impact is physical and disrupts all aspects of people’s lives, as shown in Figure 7.

In addition, the high frequency and intensity of disasters also strengthen public perception of the high level of danger in the Mount Merapi area. The eruption activity occurring almost every 2 years reflects a repeated event pattern, causing constant concern among the community. Almost all respondents stated that every time a disaster occurs, they remember it clearly because of its profound impact, which could stop all daily activities. The impact of such hazards includes structural damage as well as environmental damage. The main hazards felt by the community generally consist of primary hazards such as volcanic ash, pyroclastic flows and hot clouds, as well as secondary hazards such as cold lava floods that further increase the risk and vulnerability of the area around Mount Merapi.

In addition, some people are beginning to realise that the triggering factors for eruptions that are increasingly frequent are not only caused by natural factors but also by anthropogenic factors. In the Merapi area, there has been an increase in population density and intensity of human activities, especially in sand mining activities. Some respondents even said that eruptions are sometimes considered a profitable or happy moment for people who depend on sand mining activities for their livelihoods. All of these factors contribute significantly to the high value of hazard felt by households in the Merapi area. As shown in Figure 1, almost all households are aware of the region’s high hazard level.

Household vulnerability shows a more even distribution, as shown in Figure 8. At 44.4%, most households are in the low vulnerability category, while 30% are in the very low category. Despite this, a total of 25.6% of households still face high vulnerability, indicating a population group that is particularly vulnerable to the impact of disasters. The data highlights significant differences in resilience levels among households in the exposed regions based on social, economic, and structural vulnerabilities.

Nearly all respondents are classified as economically vulnerable because of the instability of their jobs and income sources during disasters. Some respondents even stated that they could suffer enormous losses because of disasters. The majority of the jobs affected are farmers, ranchers and similar professions, considering that most of the residents in the region work in the agricultural sector. However, there is a small number of respondents who do not experience disruption of economic stability, namely those who work as civil servants (PNS), because their income remains stable despite disasters.

Social vulnerability is related to the composition of family members based on age, vulnerability to disasters and disease conditions that can magnify the impact of disasters. The vulnerability that tends to be low in the study area is because of the dominance of the productive-age population, which is not susceptible to disasters, and the lack of cases of skin diseases or respiratory infections in the community. The productive-age population generally has high mobility, better adaptability, and more adequate access to information and resources than vulnerable groups such as children and the elderly. In addition, the low cases of diseases because of exposure to volcanic ash, such as respiratory disorders and skin irritation, highlight the physical characteristics of the community and qualified access to health. Previous studies have shown that the high incidence of the disease is highly correlated with high levels of susceptibility, as in the case of the Mount Cameroon eruption, where more than 30% of the population developed respiratory infections and more than 35% developed skin problems because of lack of protective measures and limited access to health services (Wantim et al. 2024).

**FIGURE 3:**
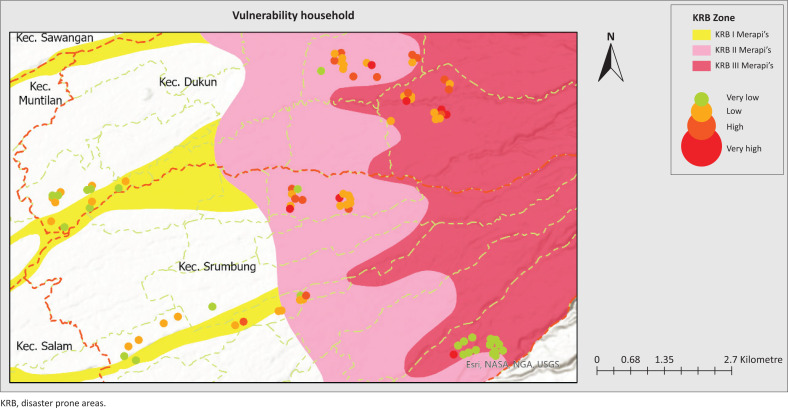
Vulnerability household mapping.

Furthermore, all buildings in the study area have been structurally adapted to local environmental conditions. The houses are constructed using locally sourced andesite stone, a material known for its high durability against volcanic hazards specific to the Merapi region. This form of structural adaptation contributes to lower levels of physical vulnerability, as illustrated in Figure 3. The reduced structural vulnerability reflects the local community’s capacity to respond to geohazard characteristics through contextually appropriate construction practices.

Household capacity to cope with disasters shows that a total of 43% of households are in the very low and low capacity category. These data indicate that almost half of the population cannot deal with the threat of disasters, making them more vulnerable to adverse impacts when disasters occur. On the other hand, a total of 52.2% of households have high and very high.

**FIGURE 4:**
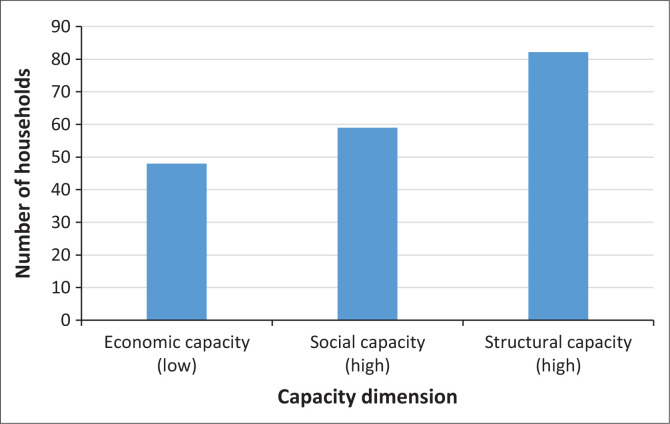
The highest value of the community perception of capacity based on households.

Figure 4 shows community capacity across three dimensions – economic, social and structural – categorised into low, middle and high levels. Economic capacity is predominantly found in the low category, reflecting the limited financial preparedness of a significant portion of respondents, particularly those residing in KRB I. These households generally lack savings or financial reserves, which reduces their ability to cope with and recover from disaster impacts. In contrast, households in KRB III exhibit higher economic capacity, often supported by stronger financial readiness. Social capacity, which encompasses access to social health insurance and participation in disaster-related activities, shows a more balanced distribution, with a noticeable increase in the middle and high categories. This indicates growing community awareness and involvement in disaster risk reduction initiatives. Structural capacity emerges as the strongest dimension, with the highest representation in the high-capacity category. This can be attributed to local adaptive practices in housing construction, where communities build resilient structures using andesite rock, a locally available material known for its durability against volcanic hazards. The data suggest that while economic preparedness remains a challenge, social engagement and structural adaptation enhancements contribute to overall community capacity. Therefore, a high level of integrated capacity can be achieved when economic, social and structural components are collectively strengthened and contextually aligned with local hazard characteristics.

## Discussion

Local spatial knowledge in this context shows that people understand their environment. In general, they can integrate spatial information from the surrounding environment into their way of thinking to identify the ability to read dangerous situations, in this case, related to the activities of Mount Merapi. One form of local understanding, namely how people monitor the hazard of their environmental conditions, is through natural signs, such as the movement of animals from highlands to lowlands, which often indicates volcanic activity. In addition, people can also distinguish the type of earthquake based on its vibration characteristics. For example, if an earthquake is felt with a pattern of vertical vibrations felt on the walls of a house, they believe it is caused by volcanic activity. Conversely, if an earthquake has a horizontal movement pattern, they assume it is derived from tectonic activity. This capability reflects high local awareness and rapid community response to hazards, which is integral to disaster mitigation strategies and aligns with research (Yasin 2014). This states the importance of social understanding in shaping vulnerability and resilience and shows that disaster management must combine the perspective and practice of the community. This adaptation pattern highlights local knowledge’s role in disaster preparedness, aligning with previous findings that traditional knowledge and rapid access to information improve preparedness for volcanic risks (Johnson et al. 2021).

However, based on Figure 1, the community has used fewer local natural signs because of technological advancement. People have tended to recognise their environmental information from technological information, like the Internet. The Internet has impacted the dissemination of information about disasters; communication technology is critical in improving community preparedness in volcanic hazard areas and enabling rapid response and preparedness for natural hazards (Gallarno et al. 2024). However, the value of local values threatened with loss needs to be maintained as a basis for disaster risk reduction. In addition, the community’s understanding of disaster-safe places, evacuation routes passed down from generation to generation, and the ability to assess the hazard of Merapi is also wisdom that can be used in mitigating the Merapi disaster.

Assessment of hazards, vulnerabilities and capacities is an essential element in community disaster risk reduction efforts. The community’s understanding of its spatial environment, formed from historical experience in facing disasters, has built awareness and adaptation to the dynamics of life in the Merapi slope area, Magelang. People in KRB III and II accept primary hazards such as lava melt, pyroclastic flows and toxic gases, and people in KRB I accept secondary hazards such as volcanic ash falling because of small eruptions. Although considered a secondary hazard, this threat cannot be ignored, given that many local practices are still in place to minimise its impact. Public awareness in volcanically hazard-prone areas is shaped by historical experience and a deep local understanding of Mount Merapi’s eruption dynamics (Lavigne et al. 2008; Mei et al. 2013).

**FIGURE 5:**
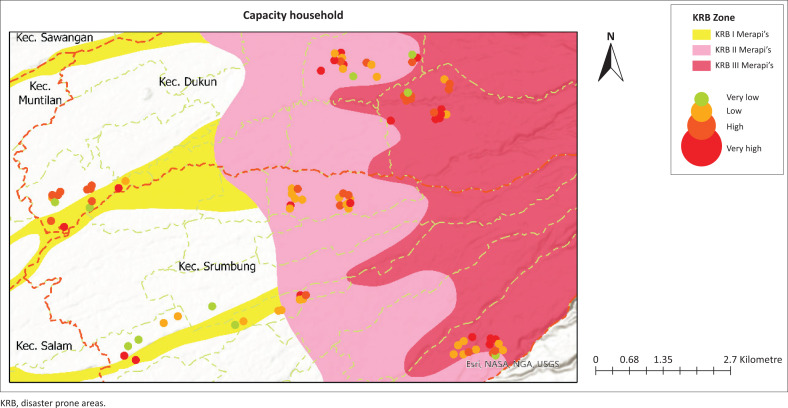
Capacity household mapping.

Community perceptions, shaped by LSK, are essential in assessing hazards, vulnerabilities and capacities for effective disaster risk management (Bostani et al. 2024; Erni et al. 2024). The interaction between these factors determines the overall level of risk society faces as capacity increases, vulnerability tends to decrease, thus reducing risk (Sultana, Tan & Hossen 2024), which highlights the inverse relationship between capacity and vulnerability in disaster contexts. This relationship is critical to developing effective disaster risk reduction strategies. Most households are in the high- and very high-risk category which are aligned with a significant level of vulnerability, especially in low-capacity households. Households with a high vulnerability, indicating that limited capacity increases the risk of the impact of existing hazards. In contrast, households with a high capacity showed a better ability to reduce risk despite being in high-risk areas. This suggests that high hazards increase household vulnerability, especially if their capacity is inadequate, while better capacity can reduce vulnerability and increase community resilience (Bergstrand et al. 2015; Paton & Johnston 2001). Therefore, disaster risk management strategies must focus on reducing vulnerability through capacity building, such as preparedness training and strengthening local resources, to create more resilient communities to disaster threats. As visualised in Figures 2, 3 and 5, spatial analysis of hazards, vulnerabilities and capacity is critical to strategically directing resource allocation and prioritising interventions in high-risk areas by combining the three assessment variables to form community resilience to the Merapi disaster.

The integration of LSK into each component of disaster analysis – hazard, vulnerability and capacity – demonstrates its operational relevance. For example, local route knowledge aids in hazard avoidance, cultural practices strengthen preparedness and traditional housing materials reflect structural capacity. These findings validate the theoretical framework of LSK as not only a conceptual tool but also an actionable tool in community-based risk reduction.

In the context of danger, LSK is manifested through community perception related to community-based observation practices such as recognising signs of eruption (e.g. increased air temperature, rumbling sounds and unusual animal behaviour). These non-instrumental indicators serve as early warning signals that complement formal monitoring systems. In addition, knowledge of natural barriers, safe paths and lava flows based on the history of previous eruptions is firmly embedded in the community’s collective memory and improves the accuracy of hazard mapping. Community perceptions help identify vulnerabilities not only to physical exposure but also to contextual social and economic weaknesses. For example, local narratives highlight that certain demographic groups, such as isolated farming families or elderly residents, face a higher risk of eruptions. These insights arise from repeated life experiences and allow communities to conduct more detailed vulnerability assessments than standard vulnerability indices. Capacity is reflected in people’s perceptions, especially traditional construction techniques (e.g. the use of andesite stones that are resistant to pyroclastic flows), community preparedness rituals and the inheritance of knowledge of evacuation routes between generations. The presence of informal alert networks and strong local leadership structures also suggests that capacity is formed through systems embedded in culture, not simply through external interventions.

**FIGURE 6:**
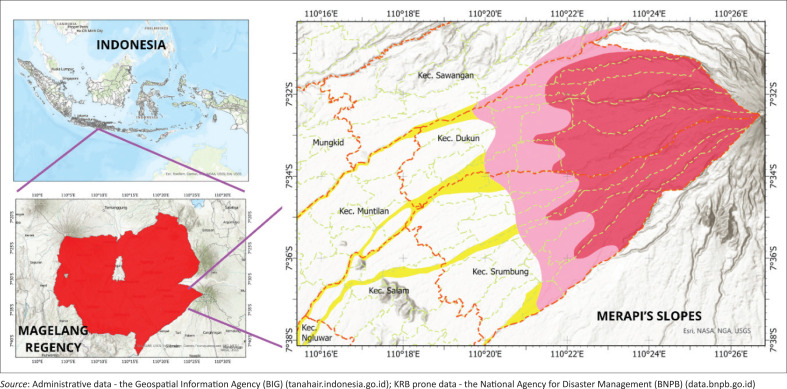
Research area.

**FIGURE 7:**
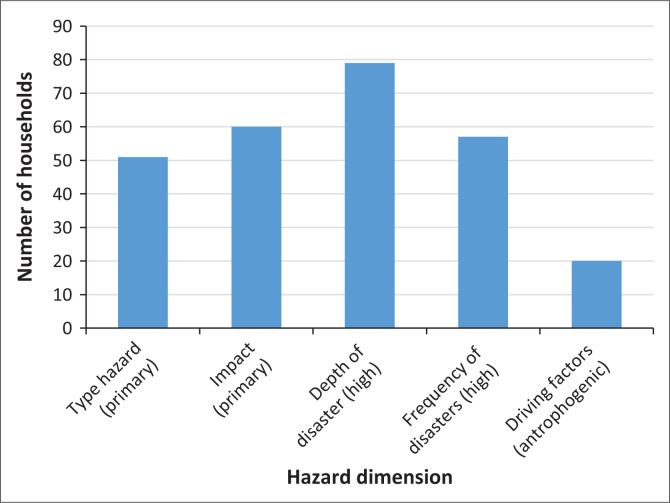
The highest value of the community perception of hazard based on households.

Tis study contributes to disaster risk reduction by demonstrating how LSK can be quantified and integrated into spatial risk analysis through PGIS. This approach not only honours community-based knowledge but also enhances risk maps’ accuracy and contextual relevance. The methodology proposed here may serve as a model for global participatory disaster planning in other volcanic-prone regions.

**FIGURE 8:**
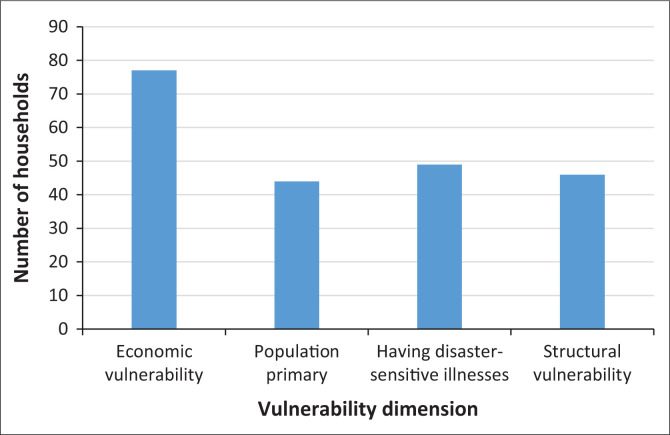
The highest value of the community perception of vulnerability based on households.

## Conclusion

Indonesia faces high geological risks, especially volcanic risk from Mount Merapi, one of the most active volcanoes in the world, with significant physical, social, economic and ecological impacts. Research shows that hazards, vulnerabilities and capacities interact with each other in determining the level of disaster risk on the slopes of Merapi, where 80% of households are in the high to very high hazard category. Low-capacity households are more vulnerable to disaster impacts, while better capacity has been shown to increase resilience. Local spatial knowledge plays a vital role in disaster mitigation, although society now relies more on modern information technology, such as digital applications. Integrating LSK and GIS provides a holistic approach to identifying hazards and vulnerabilities, as well as the capacity to assess risks and strategically direct resource allocation. By strengthening local capacity through preparedness training and resource strengthening, community resilience to the Merapi disaster can be significantly increased.

This study recommends integrating LSK with GIS technology to provide a holistic approach to identifying hazards, vulnerabilities and capacity so that it can accurately assess risks and strategically direct resource allocation. Capacity building for communities, especially low-capacity households in high-risk areas, needs to be prioritised through disaster preparedness training and modern technology such as GIS-based applications. Collaboration between governments, local organisations, educational institutions and communities is essential to create sustainable mitigation programmes. Hopefully, this LSK-based assessment can be considered for reducing disaster risk in the study area of Mt. Merapi. With the strengthening of local resources and periodic data updates, mitigation strategies can be more relevant and adaptive to increase community resilience to Mount Merapi disasters. This research is limited to exploring the assessment of hazards, vulnerabilities and capacities using LSK and the PGIS approach for visualisation.
